# Quorum sensing inhibits phage infection by regulating biofilm formation of *P. aeruginosa* PAO1

**DOI:** 10.1128/jvi.01872-24

**Published:** 2024-12-31

**Authors:** Lei Cao, Jinhui Mi, Yile He, Guanhua Xuan, Jingxue Wang, Mengzhe Li, Yigang Tong

**Affiliations:** 1 College of Life Science and Technology, Beijing University of Chemical Technology 627792 , Beijing, Beijing, China; 2 Food Safety Laboratory, College of Food Science and Engineering, Ocean University of China 506917 , Qingdao, Shandong, China; 3 Qinhuangdao Bohai Biological Research Institute, Beijing University of Chemical Technology, Qinhuangdao, Hebei, China; Michigan State University, East Lansing, Michigan, USA

**Keywords:** quorum sensing, Psl, phage adsorption, bacteria–phage interaction, *P. aeruginosa*

## Abstract

Quorum sensing (QS) can regulate diverse critical phenotypic responses in *Pseudomonas. aeruginosa* (*P. aeruginosa*), enabling bacterial adaptation to external environmental fluctuations and optimizing population advantages. While there is emerging evidence of QS’s involvement in influencing phage infections, our current understanding remains limited, necessitating further investigation. In this study, we isolated and characterized a novel phage designated as BUCT640 that infected *P. aeruginosa* PAO1. This phage belonged to class *Caudoviricetes*, genus *Bruynoghevirus*, with a podovirus morphology, and its adsorption was dependent on Psl polysaccharides, a repeating pentamer used to support biofilm structure. Leveraging phage BUCT640 as a model, we analyzed the role of both *rhl* QS and *las* QS in bacteria–phage interactions. Based on its distinctive plaque formation performances on different QS-related mutants, we investigated the variations of phage sensitivity to these strains and ultimately elucidated the mechanism underlying how QS inhibited phage infection to PAO1. Specifically, we unveiled that the *las* QS could inhibit phage adsorption, which is related to the thickness change caused by biofilm differentiation. Our findings suggest that the inhibition of QS may enhance phage infectivity, potentially facilitating advanced phage therapy combined with QS interference.

**IMPORTANCE:**

Phage therapy is a powerful solution to combat drug-resistant pathogenic bacterial infections and has earned remarkable success in clinical treatment. However, recent insights underscore the potential impact of bacterial QS on phage infection dynamics. Here, we reported a unique phenomenon wherein QS, particularly in the *las* QS pathway, showed distinctive plaque formation behaviors by enlarging halos around plaques in mutant strains. In addition to this, we first elucidated the correlation between biofilm formation and phage infection. Notably, the *las* QS could inhibit phage adsorption, an effect closely related to biofilm thickness. Such research could be the evidence to steer bacterial QS toward favorable therapeutical outcomes. Therefore, our work can extend the comprehension of the interactions between bacteria and phages influenced by QS, thereby providing new perspectives on leveraging QS interference to enhance the efficacy of phage therapy for clinical applications.

## INTRODUCTION

*Pseudomonas aeruginosa* (*P. aeruginosa*), a Gram-negative bacterium, is listed as one of the top three priority pathogens by the World Health Organization, associated with diseases such as cystic fibrosis and urinary tract infections (1–3). This Gram-negative bacterium exhibits high levels of antibiotic resistance (4), posing a significant challenge to effective treatment with existing antibiotics (5). Consequently, the exploration of alternative strategies to combat infections caused by *P. aeruginosa* has become crucial. Phage therapy, which uses viruses to target and kill bacteria specifically, has emerged as a potential alternative to antibiotic treatment. Previous cases have provided compelling evidence of the efficacy of phage therapy, such as monotherapy or in combination with antibiotics, in successfully treating multidrug-resistant *P. aeruginosa* infections (6, 7). However, bacteria have also developed multiple defense mechanisms against different stages of phage infection. For example, the modulation of receptor expression or alternation of receptors could inhibit phage adsorption (8, 9). In addition, the activation of superinfection exclusion systems serves as a mechanism to prevent phage DNA injection (10), while the utilization of restriction–modification systems and CRISPR-Cas (clustered regularly interspaced short palindromic repeats; CRISPR-associated) systems offers a means to disrupt phage DNA and subsequent replication processes (11, 12). Furthermore, abortive infection (Abi) represents a mechanism capable of inducing bacterial suicide through specific activation factors after phage entry but before completion of replication, thereby effectively inhibiting phage synthesis and release and protecting the bacterial population (13).

To counteract with phages, the expression of the anti-phage defense system in bacteria always comes with bacterial fitness costs concerning growth, metabolism, adaptation, etc. As for extracellular phage–host interactions, alternations in bacterial receptors for phages to hinder adsorption can lead to decreased bacterial fitness and virulence (14). In addition, the expression of intracellular defense elements, such as Cas proteins of the CRISPR-Cas system, can also lead to decreased competitive ability of bacterial strains (15). Given the trade-off between bacterial fitness and survival when activating defense responses, bacteria might balance their initiation by tweaking the expression by monitoring the phage pressure from the perspective of populations. Quorum sensing (QS) allows bacteria to perceive changes in the population density of the surrounding environment through specific signal molecule concentrations. Consequently, the role of QS in regulating defense responses during phage infection has gained increasing attention.

QS has been reported to regulate the expression of relevant genes to initiate specific collective behaviors, enabling adaptation to external environmental changes and maximizing the benefits of the population. Two acylated homoserine lactone (AHL)-mediated QS systems, including the *las* and *rhl* systems, widely exist in *P. aeruginosa* and control over 300 genes (16). Both systems consist of a transcriptional activator and an AHL synthase. Specifically, *lasI* directs the synthesis of N-3-oxo-dodecanoyl-homoserine lactone (3-oxo-C_12_-HSL), and *rhlI* guides the synthesis of N-butanoyl-homoserine lactone (C_4_-HSL) (17). The regulatory effects occur only if AHL signal molecules bind with the transcriptional activator. Previous evidence indicates that the QS system plays a significant role in regulating phage infection in *P. aeruginosa*, and it provides bacteria with a global regulatory defense mechanism that does not involve mutations or growth trade-offs. For example, QS involves both the modulation of phage receptor expression (18) and the regulation of the defense element system, such as the CRISPR-Cas system (19, 20). Thus, the modulation of the QS system might be considered to leverage the efficacy of phage therapy. However, our current understanding of QS on the global regulation of bacteria and phage interactions remains limited and necessitates further comprehensive investigation.

In this study, we isolated and characterized a novel phage BUCT640 infecting *P. aeruginosa* PAO1, and its adsorption was dependent on a biofilm matrix, Psl. Based on its different halo formation performances on different QS-related mutants, we investigated the variations of phage sensitivity to these strains and elucidated the mechanism underlying how QS inhibits phage infection to PAO1. Our findings can not only contribute to the understanding of the interactions between bacteria and phages influenced by QS but also provide new insights into the potential synergistic application of QS interference with phage therapy.

## RESULTS

### Characterization of phage BUCT640

**Fig 1:**
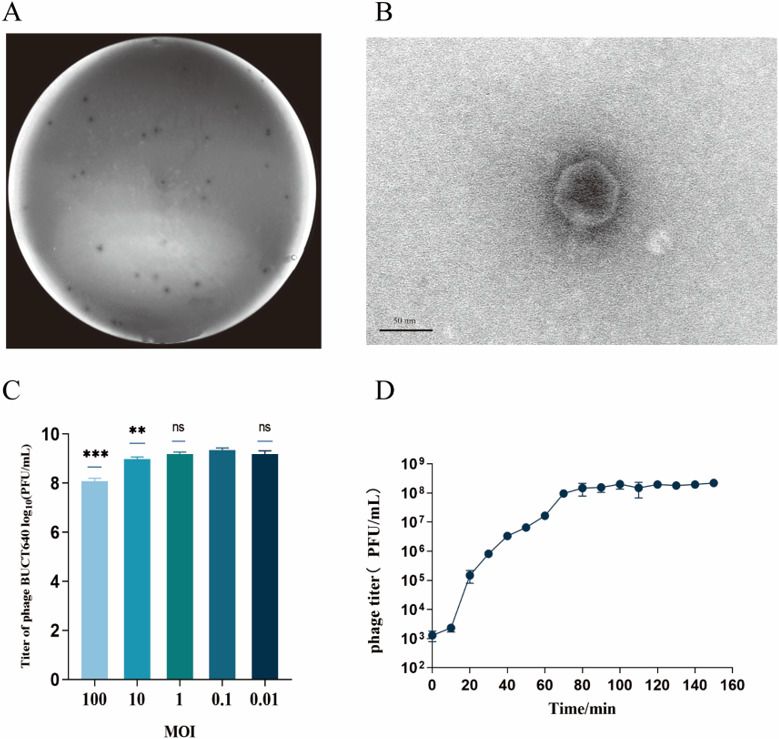
Characterization of phage BUCT640. (**A**) Plaque morphology of phage BUCT640. (**B**) TEM image of phage BUCT640. (**C**) Optimal MOI assays of phage BUCT640. (**D**) One-step growth curve of phage BUCT640 on *P. aeruginosa* PAO1. Data are shown as the mean ± SD. ***, *P*  <  0.001 and **, *P*  <  0.01 indicate a significant difference between this group and control (MOI  =  0.1), whereas ns indicates no significant difference.

A phage was newly isolated from hospital sewage using *P. aeruginosa* PAO1 as the host, and we designated it as BUCT640. Phage BUCT640 formed clear plaques on the bacterial lawn of wild-type PAO1 (Fig. 1A). Transmission electron microscopy showed that BUCT640 has a head with an approximate diameter of 63 nm and a nearly invisible short tail (Fig. 1B), revealing it to be a member of podoviruses. Phage BUCT640 was proliferated with its host under different multiplicity of infection (MOI) values, including 100, 10, 1, 0.1, and 0.01, to determine its optimal MOI. Afterward, BUCT640 reached a significantly higher final titer (3 × 10^9^ PFU/mL) at an MOI of 0.1 compared to other MOIs (*P* < 0.05), indicating that the optimal MOI for BUCT640 was 0.1 (Fig. 1C). Under the optimal MOI of 0.1, the one-step growth curve showed that the latent period of BUCT640 was approximately 10 minutes. In addition, BUCT640 reached the plateau phase at 80 minutes (Fig. 1D), with a burst size of 22 PFU/cell.

### Bioinformatics analysis of phage BUCT640

**Fig 2:**
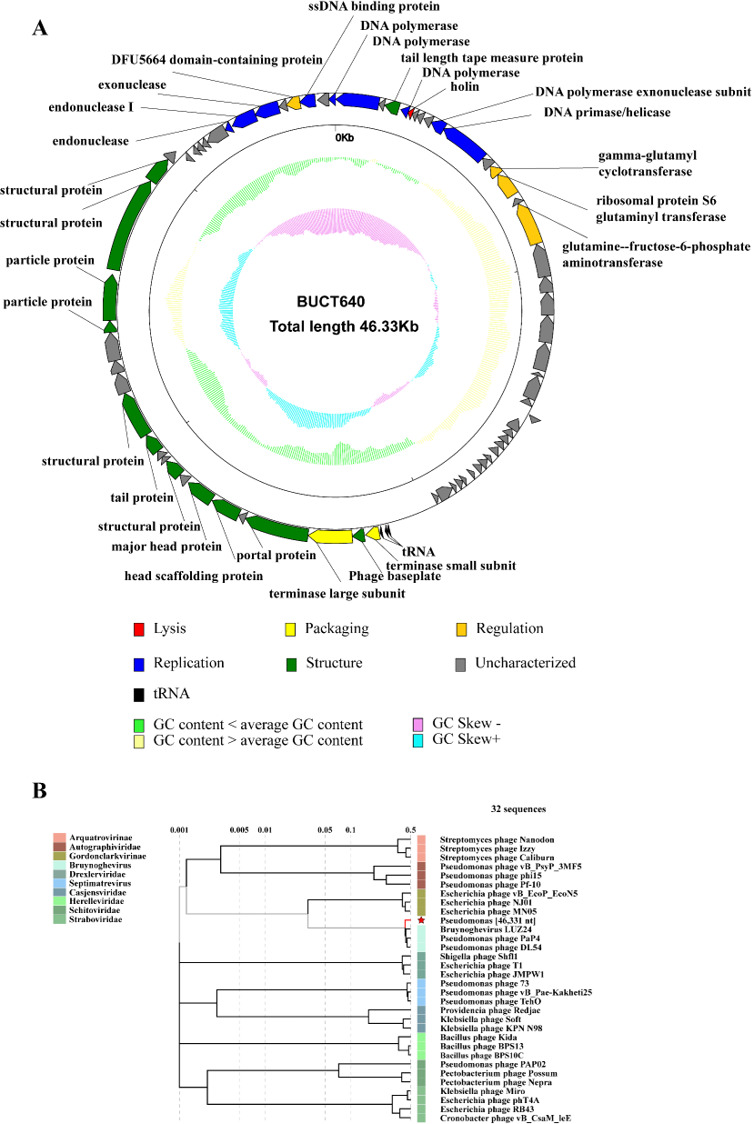
Bioinformatics analysis of phage BUCT640. (**A**) Schematic diagram of the genome of phage BUCT640. All ORFs are represented by arrows, where the positive direction is in the ring and negative direction in the inner ring. Red, lysis; yellow, package; orange, regulation; blue, replication; green, structure; gray, uncharacterized. Yellow represents greater than the average GC content, and light green represents the opposite. Light blue represents G-C/G + C  >  0, and purple represents  < 0. (**B**) Phylogenetic tree of phage BUCT640. The phylogenetic tree was constructed based on the genome sequence of BUCT640 and 31 representative phages that were homologous to phage BUCT640. Phage BUCT640 is marked with a red pentagram.

The genome of phage BUCT640 was a 46,331-bp double-stranded DNA molecule with a GC content of 52%. Based on the initial annotation with RAST and the validated annotations with BLASTp, BUCT640 had a total of 72 open reading frames (ORFs) and three tRNA genes, with 22 predicted ORFs located on the positive strand and the others on the negative strand. Bioinformatic analysis revealed that 28 ORFs encoded proteins related to phage lysis, replication, structure, and packaging, while the others were annotated as hypothetical proteins (Fig. 2A). No genes associated with virulence, resistance, or lysogenic factors were identified, confirming the lytic and nontoxic nature of BUCT640. Additionally, three types of tRNAs, including tRNA-Asn, tRNA-Asp, and tRNA-Pro, were identified between ORF38 and ORF39 in the BUCT640 genome. This suggested that phage BUCT640 could rely on its tRNA upon entering the host, facilitating the translation required for infecting other hosts and maintaining the late stage of infection (21, 22). Blastn alignment showed that BUCT640 shared the highest similarity with the *P. aeruginosa* phage oldone (GenBank accession no. MT119371.1), with the query coverage of 92% and the identity of 87.48%. Based on the ViPtree alignment results, the phylogenetic tree was constructed using 31 phages that were homologous to phage BUCT640 from different genera, indicating that BUCT640 belongs to the *Caudoviricetes* class and the *Bruynoghevirus* genus (Fig. 2B).

### Quorum sensing affects phage infection

**Fig 3:**
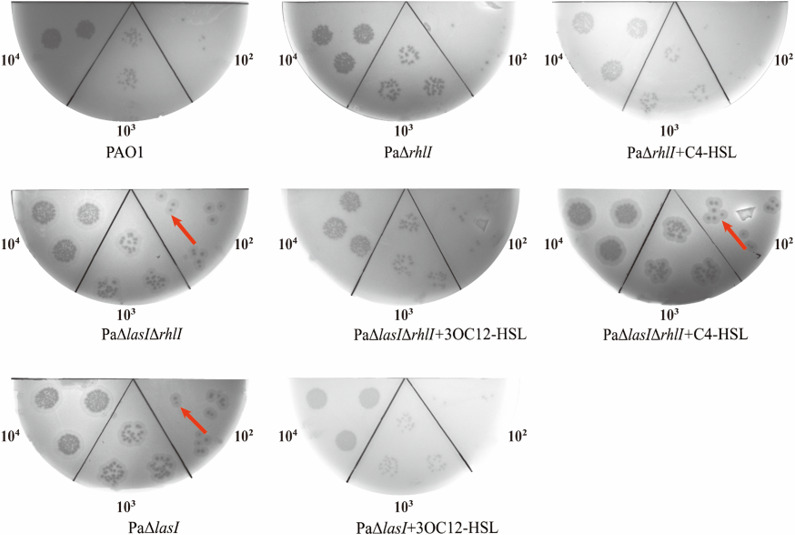
Plaque formation of phage BUCT640 on different QS mutants. Phage BUCT640 was used to infect wild-type *P. aeruginosa* PAO1 and QS mutants at phage titers ranging from 10^2^ to 10^4^ PFU/mL, with each experiment performed in triplicate. Red arrows indicate the halos formed by the phage on the lawn. To examine the effects of AHL signals on plaque morphology, C_4_-HSL was added to the PaΔ*rhlI* mutant, 3O-C_12_-HSL to the PaΔ*lasI* mutant, and both C4-HSL and 3O-C_12_-HSL were added to the PaΔ*lasI*Δ*rhlI* mutant, each at a final concentration of 5 µM.

Our objective was to understand the impact of QS on phage infection when regulating bacterial population behavior. We first observed the plaque morphology of phage BUCT640 infecting PAO1 and its different QS mutant strains, including PaΔ*lasI*, PaΔ*rhlI,* and PaΔ*lasI*Δ*rhlI*. Compared with PAO1, phage BUCT640 exhibited an enlarged halo surrounding plaques on bacterial lawns formed with PaΔ*lasI* and PaΔ*lasI*Δ*rhlI,* but PaΔ*rhlI* performed similar plaques to PAO1 (Fig. 3). This finding revealed that different plaque formation of BUCT640 on PAO1 and QS-related mutants might be caused by the variation of signaling molecules associated with *lasI*. To further validate this hypothesis, we added exogenous AHL signal molecules in the process of phage infection. Consequently, the addition of exogenous 3O-C_12_-HSL made the halo disappear in both PaΔ*lasI* and PaΔ*lasI*Δ*rhlI*, restoring the plaque morphology to the level of PAO1. However, the addition of exogenous C_4_-HSL did not induce any phenotypic changes in either PaΔ*rhlI* or PaΔ*lasI*Δ*rhlI* (Fig. 3). These results collectively indicate that the deletion of the *lasI* gene can influence phage sensitivity to *P. aeruginosa* PAO1.

**Fig 4:**
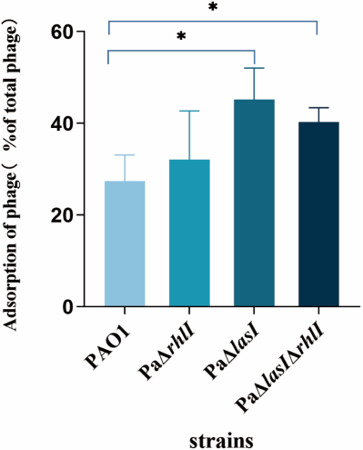
Adsorption rate of phage BUCT640 on wild-type *P. aeruginosa* PAO1 and QS mutants. Data are shown as the mean ± SD. Data are averages of three samples with standard deviations (error bars). *, *P* < 0.05 (Student’s paired *t* test).

To investigate the mechanisms associated with the change in phage susceptibility, we examined the adsorption rate of phage BUCT640 by different *P. aeruginosa* PAO1 strains. Compared to the wild-type PAO1, both Pa*ΔlasI* and Pa*ΔlasIΔrhlI* exhibited a significant increase in the adsorption rate, while Pa*ΔrhlI* did not show any difference in the adsorption rate (Fig. 4). Thus, deletion of *las* QS enhanced the adsorption rate of phage BUCT640 on *P. aeruginosa* PAO1.

### Identification of phage receptors

**Fig 5:**
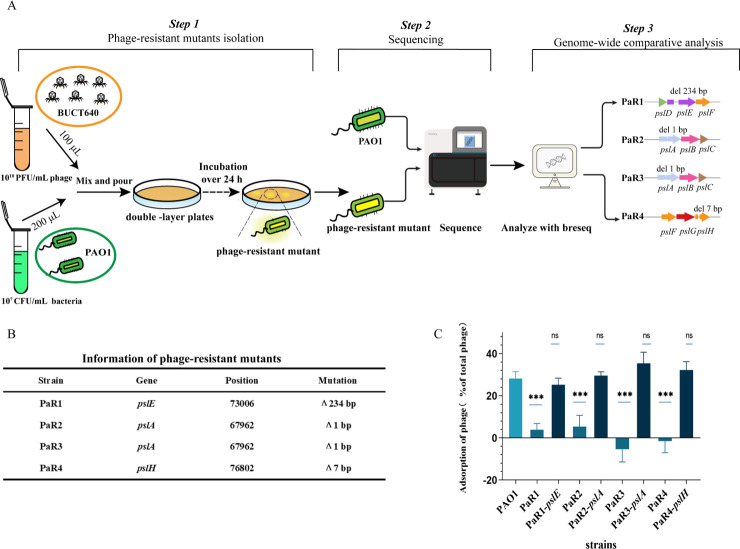
Adsorption receptor identification of BUCT640. (**A**) Schematic diagram of isolating the resistant mutant strain. Mutants were screened under high-concentration phage pressure, and mutants were sequenced and compared with the wild-type. (**B**) The detailed presentation of mutant genes. (**C**) Adsorption assays of phage BUCT640 on resistant mutants and complemented strains. Data are expressed as the mean ± SD (error bars). ***, *P* < 0.01 (paired *t* test).

To further analyze the mechanism of QS affecting phage adsorption, we identified the receptor for phage BUCT640 adsorption. We screened for four different resistant mutant strains, including PaR1, PaR2, PaR3, and PaR4, capable of surviving under phage pressures (Fig. 5A). The adsorption experiment revealed that phage BUCT640 did not adsorb to these resistant mutant strains (Fig. 5C), indicating that mutated genes among these strains might be related with the phage receptor on the bacterial surface. Genome-wide comparative analysis between wild-type PAO1 and phage-resistant mutants revealed that all resistant mutant strains had base deletions in Psl synthesis-related genes (Fig. 5B). Specifically, PaR1 had 234-bp deletion in *pslE*, PaR2 and PaR3 both had 1-bp deletion in the *pslA* gene, and PaR4 exhibited 7-bp deletion in the *pslH* gene. To further validate the role of these genes in phage adsorption, we constructed recombinant plasmids of different mutant genes and introduced them into corresponding resistant mutant strains. As shown in Fig. 5C, adsorption rates of all strains were restored to the equivalent level of the wild-type PAO1. This suggests that Psl is the receptor of phage BUCT640 on *P. aeruginosa* PAO1.

### Psl synthesis genes influence phage infection

**Fig 6:**
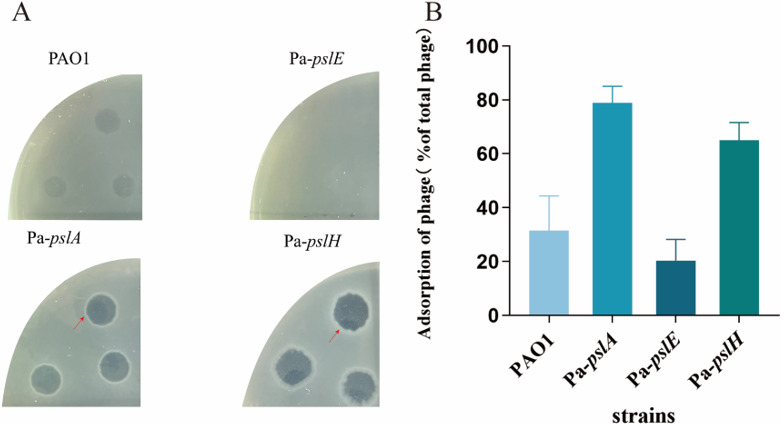
Phage plaque formation and adsorption rates on different Psl synthetic gene overexpression strains. (**A**) The plaque morphology assays of phage in wild type and Psl synthetic gene overexpression strains, including Pa-*pslA*, Pa-*pslE*, and Pa-*pslH*; the red arrows point to the halo formed by the phage on the lawn. (**B**) Adsorption rate of phage BUCT640 on wild type and Psl synthetic gene overexpression strains.

Given that the adsorption of BUCT640 is dependent on the exopolysaccharide Psl, we hypothesized that QS influences phage adsorption as well as phage plaque halo formation by modulating Psl production. Previous studies have indicated that the formation of the halo is attributed to the cleavage of bacterial polysaccharides caused by phage-derived depolymerase (23). Additionally, turbid plaques are commonly observed in high-exopolysaccharide-producing (HEP) strains. In contrast, clear plaques are observed in low-exopolysaccharide-producing (LEP) strains (24), suggesting a close association between halo performance and bacterial extracellular polysaccharides. The *las* QS affected the plaque halo formation of phage BUCT640 on bacterial lawns, and this effect is likely associated with exopolysaccharide production. Since phage BUCT640 adsorption depended on the exopolysaccharide Psl, we proposed that QS might influence phage adsorption through Psl. We overexpressed *pslA, pslE*, and *pslH* in PAO1 and then examined the plaque morphology and adsorption rate of phage BUCT640 on overexpression strains. The results showed that phage BUCT640 formed halo on both *pslA* and *pslH* overexpression strains (Fig. 6A), and the adsorption rate of the phage on the overexpression strains also increased significantly compared with that on PAO1 (Fig. 6B). For the *pslE* overexpression strain, in contrast to *pslA* and *pslH* overexpression strains, the phage adsorption rate decreased significantly (Fig. 6B), and no plaques were formed on the lawn (Fig. 6A).

### QS modulates Psl expression and biofilm formation

**Fig 7:**
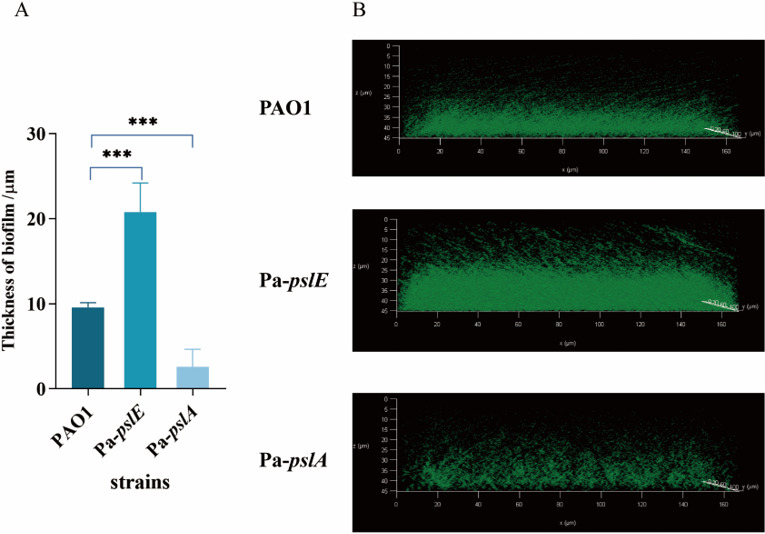
Thickness of biofilm. (**A**) Thickness of biofilms. Strains: WT, PAO1; Pa-*pslE*; Pa-*pslA*. (**B**) Laser scanning confocal photomicrographs of the WT and the Psl synthetic gene overexpression strains. Data are expressed as the mean ± SD (error bars). ***, *P* < 0.01 (paired *t-*test).

Overexpression of different Psl synthesis genes leads to different bacterial susceptibility to phage. We initially hypothesized that QS regulates bacterial susceptibility by controlling the expression of Psl synthesis genes. To test this hypothesis, we investigated the expression levels of all essential genes involved in Psl synthesis (25). In QS mutants, most genes performed downregulation ranging from onefold to fivefold (*pslA, pslC, pslD, pslE, pslF, pslG*, *pslK*, and *pslL*), while the expressions of *pslH, pslI*, and *pslJ* remained unchanged (Fig. S1). These findings indicate that QS does not uniformly regulate the expression of Psl synthesis genes. Considering the opposing effects of *pslA* and *pslE* on phage adsorption rates in overexpression strains, we speculated that other shared phenotypes may exist. Davies *et al*. reported that the absence of *las* QS system affects biofilm differentiation, while *rhlI* does not affect it. This observation aligns with those of the adsorption phenotypes, prompting further investigation. We then examined the biofilm structure of *pslA* and *pslE* overexpression strains compared to the wild-type PAO1. The results showed that the biofilm thickness formed by PAO1 was 9.6 ± 0.4 µm, while the biofilm thickness of the *pslE* overexpression strain was significantly increased to 20.8 ± 4 µm, with a denser structure (Fig. 7A and B). In contrast, the *pslA* overexpression strain had a thinner biofilm of 2.6 ± 2 µm and a significant increase in the number of planktonic bacteria (Fig. 7A and B). Therefore, we infer that a flatter biofilm makes bacteria more sensitive to phage and that the *lasI* system regulates bacterial susceptibility to phage in this way.

## DISCUSSION

QS regulates various physiological functions in bacteria, including biofilm formation, virulence factors, and motility. (17, 26, 27). Additionally, it can help bacteria overcome host immune responses and promote their survival (28). Bacterial strains with QS defects exhibit reduced competitive fitness and substantial changes in the microbial community structure and abundance (29). Accordingly, QS plays a crucial role in the physiological and ecological regulation of bacterial populations. Phages are regarded as the most abundant entities, contributing to host mortality and maintaining the diversity of their hosts in different ecological niches. Numerous recent studies indicate that apart from the role of QS on bacterial populations directly, it also involves in regulating the interaction between bacteria and phages, such as phage lysis–lysogeny switching (30, 31), and bacterial defense mechanisms against phages. Regarding the extracellular interactions, QS can modulate the number of bacterial surface receptors, thereby influencing the efficiency of phage adsorption (32, 33). In addition to this, QS can regulate the intracellular defense mechanisms to shape bacterial populations. For instance, QS can regulate the CRISPR-Cas system to prevent phage replication to survive from phage invasion, and QS can also induce transcription of critical genes of CBASS to enhance resistance against phage (19, 20, 34). We believe that the regulatory role of QS on phage and bacteria interactions is likely general and diverse beyond the detailed description here, requiring more extensive surveys.

To verify the above-mentioned hypothesis, we focused on the newly isolated phage BUCT640 and investigated its differences in plaque formation on PAO1 and QS mutant strains. Chemical complementation experiments revealed that BUCT640 formed enlarged halos surrounding plaques on the PaΔ*lasI* and PaΔ*lasI*Δ*rhlI* in comparison to wild-type PAO1 and PaΔ*rhlI*. This unique phenomenon differs from that given in previous reports on plaque transparency (18), and its mechanism remains unknown. The presence of halos around plaques has been generally considered an indicator of phage-encoded depolymerase activities (23). They can depolymerize bacterial capsular polysaccharides (CPS), exopolysaccharides (EPS), or lipopolysaccharide (LPS) on the stationary phase of the bacterial growth cycle, resulting in the formation of transparent zones known as halos (23). Given that exopolysaccharides may be regulated by QS (35), the variations in halo formation observed in *lasI*-deficient strains could be linked to changes in exopolysaccharide dynamics. Since halo formation is associated with exopolysaccharides and phage BUCT640 depended on exopolysaccharides Psl as its receptor, we hypothesized that QS may also affect phage adsorption by regulating Psl, thereby further influencing phage sensitivity. As expected, the adsorption rates of BUCT640 on both the PaΔ*lasI* and PaΔ*lasI*Δ*rhlI* strains significantly increased (Fig. 4). Our findings support those of previous reports that the presence of QS has an inhibitory effect on phage adsorption (36, 37); this regulation mode allows bacteria to resist phage infection with minimal costs and minimize the danger of autoimmunity (20). Although most recognized QS regulatory modes are negative, it is also crucial to note that QS can have a positive impact on the regulation of phage receptors. For instance, QS upregulates the expression of *galU*, the essential LPS biosynthesis gene (18). Therefore, the regulatory role of QS in the phage adsorption process is complex and diverse.

We hypothesize that QS may influence phage adsorption and halo formation by modulating Psl. However, the exact role of QS in this process remains unclear. To explore this, we first examined whether QS modulates phage susceptibility by regulating Psl gene transcription. Our analysis of Psl biosynthesis genes showed no consistent regulatory effect by QS, even for *pslA* and *pslH* (Fig. S1), which are linked to phage adsorption (Fig. 6B). This suggests that QS regulates phage susceptibility through a complex, multilayered mechanism rather than single-gene transcriptional control. Then, we attempt to identify the common principles underlying the phenotypes regulated by QS and Psl synthesis to elucidate how QS influences phage adsorption mediated by Psl formation. Previous studies have demonstrated that Psl is crucial for planktonic bacterial adhesion (38). Additionally, it has been reported that the deletion of the *lasI* system affected the process of biofilm differentiation, resulting in flatter biofilms, while the *rhlI* mutant formed biofilms similar to those of the wild-type strain (39). This was consistent with the observed adsorption phenotype, where the *lasI* system significantly impacts bacterial characteristics. Taken together, we inferred that the *lasI* QS system might alter biofilm thickness to influence phage susceptibility by regulating biofilm differentiation. To verify it, we measured the biofilm thickness of different overexpression strains and PAO1. As expected, the Pa-*pslA* strain formed flatter biofilms. (Fig. 7A and B). In contrast, the Pa-*pslE* strain developed thicker, denser biofilms and inhibited phage infection. One possible explanation for the reduced phage adsorption observed in bacteria with thicker biofilms is that the biofilm may act as a barrier, effectively hindering access of phages to their receptors (40). Another possibility is that the thick extracellular matrix and high cell densities within the biofilm inhibit phage transport via fluid bulk flow, making diffusion—a slower transport mechanism—to become predominant, thereby delaying phage adsorption and bactericidal effects (41, 42). Based on our findings, we hypothesize that an increase in biofilm thickness reduces phage adsorption, thereby diminishing the depolymerization of bacterial exopolysaccharides and ultimately inhibiting halo formation. Conversely, a decrease in biofilm thickness facilitates phage adsorption, enhances depolymerization activity, and promotes halo formation.

The adsorption to bacterial surface receptors is a crucial step in successful phage infection, and mutations related to bacterial receptors in the genome may be the simplest approach for bacteria to acquire phage resistance. However, mutations in bacterial surface phage receptors may allow bacteria to adopt trade-off strategies in adaptive costs. For example, *Lactococcus lactis* altered the gene related to the adsorption receptor, polysaccharide pellicle (PSP), to escape phage invasion and then exhibited drastic changes in cell shape and severe growth defects. These effects culminated in reduced survival rates and compromised its technological utility (43). Furthermore, the diverse array of phages with various receptor types in the environment exerts selective pressure on bacteria. If bacteria attempt to develop resistance all through mutations, the resulting tradeoff in antibiotic resistance and virulence can be unfavorable for their survival in complex natural ecosystems (44). Currently, most studies revealed that the QS system could regulate the susceptibility of bacteria, which could avoid unnecessary bacterial fitness costs resulting from receptor alternations. Stated differently, bacteria can flexibly restore their initial state upon a decrease in phage pressure, thereby ensuring their competitive advantages in various environments. In previous publications, several receptors could be regulated by QS, including LPS, outer membrane proteins, and flagella (18, 32, 33, 36, 37). To our current findings, we have expanded the understanding of QS by demonstrating its role in regulating phage adsorption by controlling biofilm differentiation through Psl, and this investigation can furnish critical evidence supporting the efficacy of phage therapy.

*P. aeruginosa* uses QS to control virulence and biofilm formation (17) so that numerous QS inhibitors (QSI) have been developed to tune or inhibit QS receptors to block bacterial pathogenesis (45). For example, as a quorum sensing inhibitor of *P. aeruginosa*, resveratrol could attenuate the pathogenicity of *P. aeruginosa* PAO1 by disturbing the TCA cycle so that anaerobic respiration could suppress the virulence (46). In addition, there are reports suggesting that QSI can be used to reduce or inhibit biofilm formation, such as benzamide–benzimidazole, baicalin hydrate, and cinnamaldehyde (47, 48). By leveraging the antivirulence and anti-biofilm-formation performances, QSIs have shown encouraging results in the clinical treatment of ventilator-associated pneumonia (49). Furthermore, our work concerning the effects of QS on bacteria and phage interaction provided positive feedback that inhibition of QS could promote phage infection. Taken together, we propose the QSI–phage combination strategy, which holds promise for the attenuation of pathogenic bacteria.

In summary, we have successfully isolated and characterized a novel phage infecting PAO1, and it belonged to class *Caudoviricetes*, genus *Bruynoghevirus*. The adsorption of BUCT640 was dependent on Psl polysaccharide, a crucial contributor to biofilm formation. Through the investigations of QS mechanisms governing bacteria and phage interactions, we have unveiled that *las* QS exerts control over the adsorption of phage BUCT640 by modulating the thickness of biofilms. While the specific regulatory pathways of QS modulating biofilm differentiation through Psl require further investigation, our finding extends the diversity of QS functions in the realm of bacteria–phage interactions and furnishes fresh perspectives on the potential combination strategy of QS manipulation with phage therapy.

## MATERIALS AND METHODS

### Bacterial strain and culture conditions

The bacterial strains and plasmid information used in this study are listed in Table S1, and the PCR primers used are listed in Table S2. All strains were cultured in Luria–Bertani (LB) medium at 37°C and stored at −80°C with 50% (vol/vol) glycerol. Gentamicin was added at a concentration of 1 mg/mL when required.

### Isolation and purification of BUCT700

Phages infecting *P. aeruginosa* PAO1 were isolated from hospital sewage samples. Sewage samples were centrifuged at 8,000 × *g* for 8 minutes, and the supernatant was filtered through a 0.22-µm filter. Next, 500 µL of the filtrate was mixed with 500 µL of *P. aeruginosa* PAO1, and the mixture was added to 5 mL of the LB broth and incubated at 37°C with shaking. After overnight incubation, the mixture was centrifuged at 12,000 × *g* for 3 minutes, and the supernatant was filtered through a 0.22-µm filter to discard bacteria. Afterward, phages were isolated using a double-layer plate method (50). Briefly, the soft agar containing the host strain and suspected phages were plated on a bottom agar plate to form host lawns and allow for plaque formation. Then, the individual plaques were picked up and suspended in phosphate-buffered saline (PBS) for proliferation and purification. The purification was conducted at least three times. The purified phages were stored at 4°C and stored at −80°C in 20% glycerol.

### Electron microscopy

Phage morphology was observed using a previously described method with minor modification (50). High-titer phages were prepared using density-gradient ultracentrifugation. In brief, 30 mL of the phage lysate was centrifuged at 8,000 × *g* for 10 minutes, and the supernatant was filtered using a 0.22-µm filter. The supernatant was then added to a 50-mL ultracentrifuge tube (Beckman Coulter, USA), and 30% (wt/vol) sucrose solution was slowly injected into the bottom of the tube. Next, the mixture was centrifuged at 35,000 × *g* for 2 hours at 4°C, and the concentrated phages were resuspended in 200 µL PBS. Finally, purified phages were negatively stained with 2% (wt/vol) phosphotungstic acid (pH  =  7.0) for 2  minutes and examined using a JEM-1200EX transmission electron microscope at an acceleration voltage of 100 kV.

### Optimal multiplicity of infection (MOI) determination

To determine the optimal MOI of phage BUCT640, the mixture of BUCT640 and *P. aeruginosa* PAO1 at different MOIs (100, 10, 1, 0.1, and 0.01) was introduced into 10  mL of LB broth. This mixture was then subjected to overnight incubation at 37°C with shaking at 200  rpm. Then, the phage titer was determined by the double-layer plate method, and the MOI with the highest phage titer was the optimal MOI. The experiments were performed in triplicate.

### Genomic DNA extraction, sequencing, and bioinformatic analysis

Phage DNA was extracted using the phenol–chloroform extraction method (50). The NEBNext Ultra II DNA Library Prep Kit for Illumina (#E7645) was used for phage genomic library construction. The phage genome was sequenced by NovaSeq 6000 (Illumina, San Diego, USA) using NovaSeq 6000 S4 Reagent Kit v1.5 (Illumina, San Diego, USA) with a PE150 sequencing strategy. Spades v3.13.0 (http://cab.spbu.ru/software/spades/) was used to assemble the filtered data (51). Putative open reading frames (ORFs) in this phage genome were predicted using the online tool RAST (https://rast.nmpdr.org/rast.cgi) (52). All predicted ORFs were annotated against the nonredundant protein database (NR) in the National Center for Biotechnology Information (NCBI) (http://blast.ncbi.nlm.nih.gov/). The genome map was constructed using internal Python scripts. A phylogenetic tree based on the complete genome sequences of 31 representative phages homologous to phage BUCT640 was built using VIPtree (https://www.genome.jp/viptree/) with BUCT640 as the reference genome (53).

### One-step growth curve

*P. aeruginosa* PAO1 was infected with phage BUCT640 under the optimal MOI of 0.1. After initial incubation at room temperature for 10 minutes, the mixture was centrifuged at 12,000 × *g* for 5 minutes. The resulting supernatant was discarded, and the precipitate was resuspended and washed with 1 mL of LB broth. Subsequently, the suspension was added to 40 mL of LB broth and incubated at 37°C with shaking at 200 rpm. Samples were taken at 10-minute intervals for 150 minutes, followed by centrifugation at 12,000 × *g* for 2 minutes. The phage titer was determined via the soft agar overlay method, and the burst size and latent period could be obtained from the one-step growth curve.

### Phage susceptibility assay for PAO1 and its mutants

To test the susceptibility of *P. aeruginosa* PAO1 and its QS-related mutants to the isolated phage, bacterial hosts were grown in 5 mL LB broth to reach an OD_600_ of 1.0. Phage lysates were diluted to prepare a series of tenfold dilutions ranging from 10^9^ to 10^2^ PFU/mL. Then, 200 µL of the bacteria culture was mixed with 5 mL of 1% melted agar and LB broth to prepare double-layer plates. For signal molecular complementation experiments, AHL signal molecules were dissolved in dimethyl sulfoxide (DMSO) and added to the melted 1% agar and LB medium to obtain a double-layer plate at a final concentration of 5 µM. As a control, an equivalent volume of DMSO was added as a blank control. The diluted phages were then spotted onto a plate and incubated at 37°C for 12 hours. Plates were imaged with Tanon 5200 multi-imaging system (Tanon, China).

### Adsorption rate assay

To determine the adsorption rate of phages on different bacterial strains, single colonies of PAO1 and its QS-related mutants were respectively cultured overnight in LB broth (OD_600_ = 1.0). Then, 900 µL of the bacterial suspension was mixed with 100 µL of the phage lysate (10^4^ PFU/mL), and the resulting mixtures were incubated at 37°C for 8 minutes, followed by centrifugation at 4°C and 12,000 × *g* for 3 minutes. The titers of un-adsorbed free phages in the supernatant were determined using a double-layer plate method (50). The adsorption rate of phages was calculated as follows: Adsorption rate (%) = [(initial phage titer - unadsorbed free phage titer in the supernatant) / (initial phage titer)] ×100%.

### Preparation and identification of phage-resistant mutants

To obtain phage-resistant mutants, we employed a previously described method with a minor modification (54). In brief, PAO1 was mixed with phage BUCT640 at an MOI of 100 in LB broth and cultured overnight at 37°C with shaking. The mixture was then centrifuged at 4°C and 12,000 × *g* for 5 minutes, and the precipitate was resuspended in 1 mL of LB broth. Next, 100 µL of the resuspended cells was streaked on LB agar plates supplemented with a high concentration of phages (10^9^ PFU/mL) and incubated overnight at 37°C. Under phage selective pressures, survival colonies were then picked and grown in fresh LB broth for subsequent analysis. To examine their phage resistance, different dilutions of phages were spotted onto bacterial lawns formed on the soft agar and placed at 37°C for 8 hours. Afterward, genomic DNA of PAO1 and phage-resistant mutants with impaired adsorption were both extracted using the Bacterial Genomic DNA Extraction Kit (Solarbio, China) and sequenced in Novogene Bioinformatics Technology Co., Ltd. The clean data were assembled using SPAdes v3.13.0, and mutations present in resistant strains were analyzed using breseq v0.37.1 (55).

### Identification of phage receptor-associated genes

Based on mutations of phage-resistant mutants, a complementation experiment was performed to identify phage receptor-associated genes using the previously described method with modifications (56). The Psl synthesis genes were amplified from *P. aeruginosa* PAO1 chromosomal DNA by PCR. The amplicons were digested with restriction enzymes EcoRI and BamHI and then cloned into the plasmid pUCP24 to construct the recombinant plasmids. Subsequently, the recombinant plasmids were then transformed into phage-resistant mutants by electroporation (2.5 kV, 200 Ω, 25 µF, 5 seconds). Bacterial lawns formed by transformants in the soft agar supplemented with 20 µg/mL gentamicin were used for phage adsorption as mentioned above.

### Gene overexpression

To verify the effects of Psl synthesis genes to bacterial susceptibility, different Psl synthesis genes were overexpressed in wild-type PAO1. The Psl synthesis genes were amplified from *P. aeruginosa* PAO1 chromosomal DNA by PCR. The amplicons were digested with restriction enzymes EcoRI and XbaI and then cloned into the plasmid pHB20T to construct the recombinant plasmids. Subsequently, the recombinant plasmids were then transformed into PAO1 by electroporation (2.5 kV, 200 Ω, 25 µF, 5 seconds). After electroporation, cells were incubated at 37°C for 1 hour. The activated cells were plated on LB agar containing gentamicin (20 µg/mL), and single colonies were cultured in LB broth at 37°C until the exponential phase. Following this, 20% (wt:vol) arabinose (10 µL/mL) was added to induce gene expression. Adsorption rate assay and phage sensitivity assay were performed using the aforementioned method.

### RT-qPCR

Single colonies of PAO1 and its QS-mutants were cultured in LB broth at 37°C with shaking overnight, and cells were harvested at the OD_600_ of 1.5. RNA was extracted using the Flying Shark Plus Bacteria RNA kit (Noblebay, China). cDNA was synthesized using the HiScript II Reverse Transcription Kit (Vazyme, China). Real-time quantitative reverse transcription-PCR (RT-qPCR) was performed using *Taq* Pro Universal SYBR qPCR Master Mix (Vazyme, China), heat-labile uracil-DNA glycosylase (UDG) (Vazyme, China), and deoxyuridine triphosphate (dUTP) (Vazyme, China). The relative expression levels of target genes were calculated using *rplS* as the reference gene.

### Confocal laser scanning microscopy

Single colonies of PAO1 and overexpression strains were respectively cultured overnight in LB broth to an OD_600_ of 1.0. Then, 10 µL of culture and 990 µL LB broth were transferred into confocal dishes (NEST, China). In the overexpression strains, 10 µL 20% (wt:vol) arabinose was added, while 10 µL of saline solution was added to the PAO1 culture. The cultures were then incubated at 37°C for 24 hours. After incubation, the medium was removed, and the planktonic cells were washed with saline. The biofilms were stained with SYTO 9 for 30 minutes in the dark, followed by washing in saline. Biofilm structures were visualized using a laser confocal microscope (Leica, Germany) equipped with 10 × eyepiece × 10×objective lens, with Ex/Em settings of 480/500 nm. Thickness of the biofilm was measured according to the scale given by Las X software.

### Statistic statements

Data were expressed as means ± standard deviation. *P* values were calculated using Student’s *t*-test, except for data in Fig. 1C, which were analyzed using one-way ANOVA. Analysis was carried out using GraphPad Prism v.8 software.

## Data Availability

The complete genome sequence of phage BUCT640 has been deposited in GenBank under the accession number OR000396. The sequencing data of *P. aeruginosa* PAO1 and phage-resistant mutant strains were submitted to the NCBI database under the accession numbers SAMN36965448, SAMN43603727, SAMN43603728, SAMN43603729, and SAMN43603730.

## SUPPLEMENTAL MATERIAL

Supplemental material jvi.01872-24-s0001.docx Tables S1 and S2; Figure S1.

ASM does not own the copyrights to Supplemental Material that may be linked to, or accessed through, an article. The authors have granted ASM a non-exclusive, world-wide license to publish the Supplemental Material files. Please contact the corresponding author directly for reuse.
