# Supplementary material for: Quorum sensing inhibits phage infection by regulating biofilm formation of P. aeruginosa PAO1
Source: J Virol. 2024 Dec 31;99(2):e01872-24. doi: 10.1128/jvi.01872-24 (PMC11853092; doi:10.1128/jvi.01872-24)
Supplement: Supplemental material — Tables S1 and S2; Figure S1. [file jvi.01872-24-s0001.docx]

**Supporting information for**

**Quorum sensing inhibits phage infection by regulating biofilm formation of *Pseudomonas aeruginosa* PAO1**

Lei Cao^a^, Jinhui Mi^a^, Yile He^a^, Guanhua Xuan^b^, Jingxue Wang^b^, Mengzhe Li^a^*, Yigang Tong^a^*

^a^ College of Life Science and Technology, Beijing University of Chemical Technology, Beijing, China

^b^ Food Safety Laboratory, College of Food Science and Engineering, Ocean University of China, Qingdao, China;

Address correspondence to Mengzhe Li, futurelmz123@163.com, and Yigang Tong, [yigang@gmail.com](mailto:yigang@gmail.com).

**Table S1 Strains and plasmids used in this study.**

| **Strain/plasmid** | **Description** | **Source** |
| --- | --- | --- |
| ***Escherichia coli* strains** |  |  |
| DH5ɑ | Cloning strain | Solarbio |
| ***P. aeruginosa* strains** |  |  |
| PAO1 | Wild type | Xuan G H^a^ |
| PaΔ*lasI* | PAO1 mutant with *lasI* genes disrupted | Xuan G H |
| PaΔ*rhlI* | PAO1 mutant with *rhlI* genes disrupted | Xuan G H |
| PaΔ*lasI*Δ*rhlI* | PAO1 mutant with *lasI* and *rhlI* genes disrupted | Xuan G H |
| PaR1 | PAO1 mutant resistant phage BUCT640 | This study |
| PaR2 | PAO1 mutant resistant phage BUCT640 | This study |
| PaR3 | PAO1 mutant resistant phage BUCT640 | This study |
| PaR4 | PAO1 mutant resistant phage BUCT640 | This study |
| PaR1-*pslE* | *pslE* complementary PaR1 strain | This study |
| PaR2-*pslA* | *pslA* complementary PaR2 strain | This study |
| PaR3-*pslA* | *pslA* complementary PaR3 strain | This study |
| PaR4-*pslH* | *pslH* complementary PaR4 strain | This study |
| Pa-*pslA* | *pslA* gene overexpression in PAO1 | This study |
| Pa-*pslE* | *pslE* gene overexpression in PAO1 | This study |
| Pa-*pslH* | *pslH* gene overexpression in PAO1 | This study |
| ***Plasmids*** |  |  |
| pUCP24 | \| GmR, ori, lacZα, Rep, oriV \| \| --- \| | Le S^b^ |
| pUCP24-*pslA* | pUCP24 contain *pslA* | This study |
| pUCP24-*pslE* | pUCP24 contain *pslE* | This study |
| pUCP24-*pslH* | pUCP24 contain *pslH* | This study |
| pHB20T | GmR, ori, lacZα, Rep, oriV, araBAD promoter | Le S |
| pHB20T-*pslA* | pHB20T contain *pslA* | This study |
| pHB20T-*pslE* | pHB20T contain *pslE* | This study |
| pHB20T-*pslH* | pHB20T contain *pslH* | This study |

^a^ Xuan G H. Food Safety Laboratory, College of Food Science and Engineering, Ocean University of China.

^b^ Le S. Department of Microbiology, College of Basic Medical Sciences, Key Laboratory of Microbial Engineering Under the Educational Committee in Chongqing, Army Medical University.

**Table S2 Primers used in this study.**

| **Primers** | **Sequence (5'-3')** | **Usage** |
| --- | --- | --- |
| pslA-F | ATGCATTCGAAGTCGGTAGATAG | Primers for RT-qPCR |
| pslA-R | GTCCAGTGCCTGGAACATAA |  |
| pslC-F | CCTGTATCGCGTACCTGAAAT | Primers for RT-qPCR |
| pslC-R | CTTCCAGTAGCCTGGAAACAT |  |
| pslD-F | TCGCCACCATCTATGAACTG | T Primers for RT-qPCR |
| pslD-R | TCAGCTCGTTGGCGATTT |  |
| pslE-F | CAAGAGCCAGATCAGCAACA | Primers for RT-qPCR |
| pslE-R | CGAGCAGGTAGCAGAGATAGA |  |
| pslF-F | GAGTCGAAGAAACTCGGTCTG | Primers for RT-qPCR |
| pslF-R | TTCAACAACTGTTCGGAGAGG |  |
| pslG-F | CAGCTGAAGTCGGTGTTCTAC | Primers for RT-qPCR |
| pslG-R | GGTTGGGCTCGTTCCATAC |  |
| pslH-F | GAACGCCGGGTGATGAG | Primers for RT-qPCR |
| pslH-R | GTATTCGTAGTTGCCGAGGAA |  |
| pslI-F | ACTACCTGTCGATCGCCTAT | Primers for RT-qPCR |
| pslI-R | GAACCAGGGCACGTTCTT |  |
| pslJ-F | CCTGTTCGACCACTACTTCAG | Primers for RT-qPCR |
| pslJ-R | TCGCTTGAGCTTGCGATAA |  |
| pslK-F | TGATGCTCCATGCGCTATTC | Primers for RT-qPCR |
| pslK-R | GCCAGGGCCAGTTGTTC |  |
| pslL-F | GGCATCCTGTTCCTCAAGAC | Primers for RT-qPCR |
| pslL-R | CAGGACATAGCCGAACATGAA |  |
| rplS-F | ATACCGTGATCGTCCAGGTC | Primers for RT-qPCR  Primers for verify of recombinant plasmid pUCP24 |
| rplS-R | GGCTGTAGGTCTGGAAGGTA |  |
| Check24F | AGGCGATTAAGTTGGGTAACG |  |
| Check24R | TTTATGCTTCCGGCTCGTATG |  |
| 24E-F  24E-R | TATGACCATGATTACGAATTCATGATAGAAATTCGTTCCTTGCG  CAGGTCGACTCTAGAGGATCCTCAGAACGCGCTCCGGTA | Primers for construction of pUCP24-*pslE* |
| 24A-F | TATGACCATGATTACGAATTCATGCATTCGAAGTCGGTAGATAGC | Primers for construction of pUCP24-*pslA* |
| 24A-R | TGCCTGCAGGTCGACTCTAGATCAGTAGACTTCCTTGGTCAGGAGT |  |
| 24H-F | TATGACCATGATTACGAATTCATGCGTATTCTCTGGATCCTGC | Primers for construction of pUCP24-*pslH* |
| 24H-R | TGCCTGCAGGTCGACTCTAGACTATGCGCATGCCGGCGC |  |
| Check20F | CCATAAGATTAGCGGATCCTACC | Primers for verify of recombinant plasmid pHB20T |
| Check20R | TGTGCTGCAAGGCGATTA |  |
| 20A-F | AACGATGGCGATTGCGAATTCATGCATTCGAAGTCGGTAGATAGC | Primers for construction of pHB20T-*pslA* |
| 20A-R | TGCCTGCAGGTCGACTCTAGATCAGTAGACTTCCTTGGTCAGGAGT |  |
| 20E-F | AACGATGGCGATTGCGAATTCATGATAGAAATTCGTTCCTTGCG | Primers for construction of pHB20T-*pslE* |
| 20E-R | TGCCTGCAGGTCGACTCTAGATCAGAACGCGCTCCGGTA |  |
| 20H-F | AACGATGGCGATTGCGAATTCATGCGTATTCTCTGGATCCTGC | Primers for construction of pHB20T-*pslH* |
| 20H-R | TGCCTGCAGGTCGACTCTAGACTATGCGCATGCCGGCGC |  |


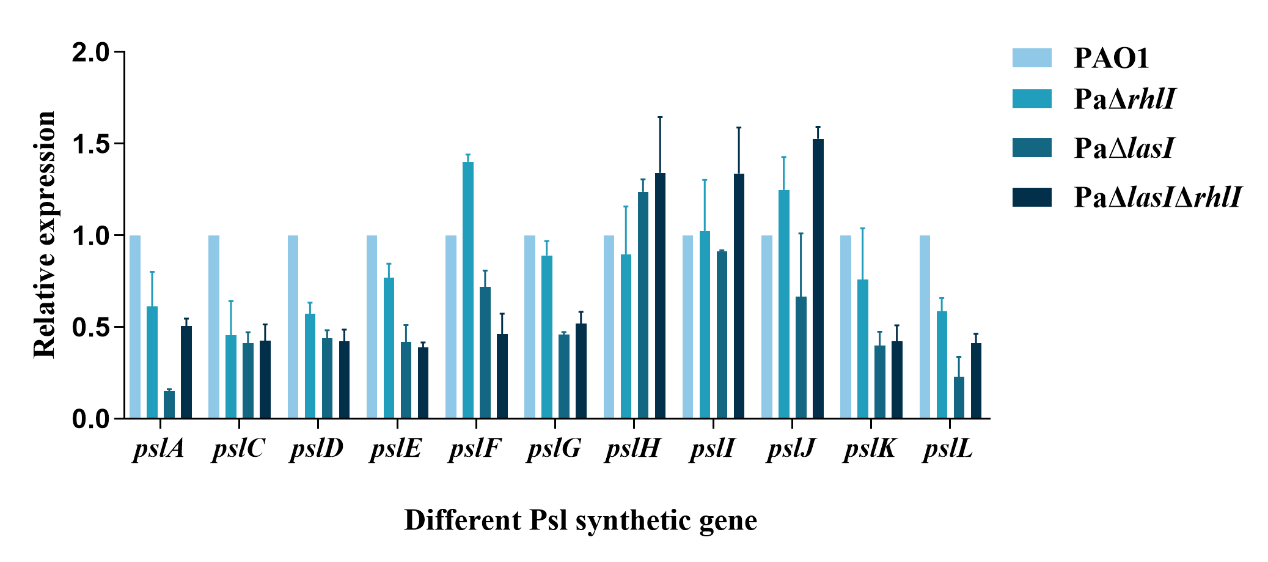


**Fig. S1** Relative expression of Psl-related genes in wild-type PAO1 and its QS mutants. The reference gene was *rplS*. Data were expressed as mean ± standard deviations.
